# Enhanced Controlled Transdermal Delivery of Mexazolam Using Ethylene-vinyl Acetate

**Published:** 2012

**Authors:** Cheong Weon Cho, Sang Chul Shin

**Affiliations:** a *College of Pharmacy, Chungnam National University, Daejeon, 305-754, Korea.*; b *College of Pharmacy, Chungnam National University, Gwanju 500-757, Korea.*

**Keywords:** Mexazolamm, EVA, Matrix, Plasticizer, Transdermal delivery, Penetration enhancer.

## Abstract

Repeated oral administration of mexazolam, an anti-anxiety agent, may cause adverse effects such as gastric disturbance, drowsiness, and ataxia due to transiently high blood levels. Transdermal administration would avoid the systemic side effects and gastric disorders after oral administration. We have developed a matrix using ethylene-vinyl acetate (EVA), a heat-processible and flexible material, for transdermal delivery of mexazolam. Drug solubility was highest at 40% PEG-400 volume fraction. The release and permeation profiles through the rat skin were determined for 24 h using a modified Keshary-Chien diffusion cell. The drug release was increased by increasing the concentration with a linear relationship between the release rate and the square root of loading dose. Increasing temperature increased drug release from the EVA matrix. The activation energy (E_a_), which was measured from a slope of log P versus 1000/T plot, was 8.64 Kcal/mol for a 1.5% loading dose. To reduce the brittleness and increase the pore of the EVA matrix, diffrent plasticizers were used. Among the plasticizers, including the citrates or the phthalate groups, diethyl phthalate showed the highest effect on the release of mexazolam. To increase the skin permeation of mexazolam from the EVA matrix, enhancers such as the fatty acids, the pyrrolidones, the propylene glycol derivatives, the glycerides, and the non-ionic surfactants were added to the EVA matrix, respectively, and skin permeation was evaluated using a modified Keshary-Chien diffusion cell fitted with intact excised rat skin. Among the several enhancers used, *N*-methyl-2-pyrrolidone showed the best enhancement factor. In conclusion, enhanced transdermal delivery of mexazolam through an EVA matrix containing plasticizer and a permeation enhancer could be useful in the development of a transdermal drug delivery system.

## Introduction

Generally, oral administration of mexazolam, an anti-anxiety agent, may cause adverse effects such as drowsiness, and ataxia due to transiently high blood levels. Transdermal delivery can avoid the systemic side effects and gastric disorders after oral administration. Transdermal drug delivery uses the skin as an alternative route for the delivery of systemically acting drugs and has several advantages over oral drug administration. Transdermal administration circumvents the variables that can influence gastro-intestinal absorption such as pH, food intake, and gastro-intestinal motility. It bypasses the hepatic metabolism and is therefore suitable for drugs with a low bioavailability. Transdermal drug delivery can give a constant and controlled drug input and decreases the variations in drug plasma levels, thus reducing the side effects, particularly of drugs with a narrow therapeutic window. Despite the many advantages of the skin as a site of drug delivery, few drugs can penetrate the skin, primarily because of the low permeability of drugs in the stratum corneum, the outermost layer of the skin. The highly organized crystalline lipid lamellae play an essential role in the barrier properties of the stratum corneum. A penetration enhancer can circumvent the barrier function of the skin by increasing the permeability of the stratum corneum (1). 

Ethylene vinyl acetate (EVA) copolymer, a member of the polyolefin family derived from random copolymerization of vinyl acetate and ethylene, is a heat-processible, flexible, and inexpensive material used for transdermal drug delivery. We performed this study to determine the feasibility of transdermal delivery of mexazolam by studying the characteristics of its in vitro release. We tested the effect of temperature as well as adding plasticizers and penetration enhancers on drug permeability in rat skin. To increase the skin permeation of mexazolam, penetration enhancers were added to the EVA matrix system and the level of mexazolam permeation through rat skin was evaluated. The aim of this study was to develop an EVA matrix system containing a permeation enhancer for the transdermal delivery of mexazolam.

## Experimental

Mexazolam was supplied from Hanhwa Pharm. Co., Ltd. (Korea). Ethylene vinyl acetate (EVA, 40%) was purchased from Aldrich Chemical Co., Inc. (St. Lois, MO, USA); polyethylene glycol was obtained from Yakuri pure chemicals Co. Ltd. (Japan). Acetyl tributyl citrate, acetyl triethyl citrate, tributyl citrate, and triethyl citrate were purchased from Morflex, Inc. (USA). Diethyl phthalate and di-n-butyl phthalate were from Junsei Chemical Co., Ltd. (Japan). PEG 400 and chloroform were from Daejung Chemical & Metals. Co. Ltd. (Korea). Lauric acid, oleic acid, and caprylic acid were purchased from Tokyo Kasei Kogyo Co., Ltd (japan). 2-pyrrolidone was obtained from Acros organics (USA). Myristic acid, linoleic acid, 1-methyl-2-pyrrolidone, polyoxyethylene-2-stearyl ether (Brij 72), polyoxyethylene-23-lauryl ether (Brij 35) and polyoxyethylene-2-oleyl ether (Brij 92) were purchased from Sigma-Aldrich Co. (St. Louis, MO, USA). Macrogol-6 glycerides, caprylocaproyl mcarogol-8 glycerides, propylene glycol laurate, and propylene glycol monolaurate were gifts from Gattefose (St. Priest, France). Stearic acid was bought from Hayashi Pure Chemical Industries Ltd. (Japan) and palmitic acid was purchased from Kanto Chemical Co. Inc. (Japan). Acetonitrile was HPLC grade from J.T.Baker Inc. (USA). All reagents were of analytical grade and were used without further purification. 

*Determination of drug solubility*

Excess amounts of mexazolam were equilibrated with saline containing various concentration of PEG 400. Each solution was shaken at 37°C for 24 h in a shaking incubator. The solutions were then filtered through a 0.45 μm filter membrane. The concentration of mexazolam was determined at 242 nm by UV spectrophotometer after dilution. 

*Permeation of mexazolam through the EVA membrane *

About 2 g of EVA copolymer beads was dissolved in 20 mL of chloroform in a glass beaker. This polymer solution was poured onto a glass plate and the solvent was allowed to evaporate at room temperature overnight. The membrane was removed from the plate. A piece of membrane was then cut properly and the thickness (120 μm) was measured before the experiment. 

For the determination of steady state permeation of mexazolam through the EVA membrane, a two chamber-diffusion cell was used. Each half-cell has a volume of about 7 mL and an effective diffusion area of 0.79 cm^2^. A piece of EVA membrane was clamped between the two halves of the cell and the assembled cell was placed in a shaking incubator at 37°C. A drug suspension in various concentrations of PEG 400 solution was added to the donor compartment, and the same concentration of PEG 400 solution (without the drug) was added into the receptor compartment, to prevent the effect of solvent permeation. The cell was shaken horizontally at 150 rpm to minimize the boundary effect. The total volume of the receptor solution was removed at the predetermined intervals and replaced with 7 mL of fresh solution. The amount of permeated drug was determined at 242 nm by UV spectrophotometer. 

*In-vitro release study*

*Preparation of drug-EVA matrix containing plasticizer*

Drug-EVA matrix containing plasticizer was prepared by casting. A weighed amount of EVA copolymer beads was dissolved in 20 mL of chloroform in a beaker and the drug solution was added. Plasticizer was dropped into the drug-containing EVA solution and mixed for 1 h. This method was chosen to produce large unharmed pieces of the membrane with no specific molecular orientation. This mixture was poured onto a glass plate and the solvent was allowed to evaporate at room temperature overnight. Plasticizer was added at 5% (w/w) of the EVA matrix. Alkyl citrates such as acetyl tributyl citrate (ATBC), tributyl citrate (TBC), acetyl triethyl citrate (ATEC), or triethyl citrate (TEC), as well as phthalates such as diethyl phthalate (DEP) and di-n-butyl phthalate (DBP) were used as plasticizers. 

*In-vitro release studies from the drug-EVA matrix*

The *in-vitro* release of mexazolam from the EVA matrix was examined by using a modified Keshary-Chien cell. A unit of the EVA matrix was clamped between the cell cap and receptor cell. The diameter of the cell was 2 cm, providing 3.14 cm^2^ of constant area between the matrix and the bulk solution of 21 mL. The receptor, 40% PEG 400 solution, was maintained at 37°C with a circulating water jacket and stirred constantly at 350 rpm. Before the experiment, the system was tested to remove the remaining air bubbles in the receptor site. At predetermined time intervals, the whole solution from the receptor cell was taken and replaced with fresh solution. The cumulative amount of mexazolam released from the matrix was determined at 242 nm by UV spectrophotometer. The effects of drug concentration on its release from the EVA matrix were studied at concentrations of 0.5%, 1%, 1.5%, 2%, and 2.5% (w/w). The effect of temperature on drug release was studied at 27, 32, 37, and 42 °C. Each data point represents the average of three determinations. 

The enhancement factor (EF) was calculated using the following equation: 

EF = (flux of EVA matrix containing plasticizer) / (flux of control sample) 

*In-vitro skin permeation study from EVA matrix through rat skin*

*Skin preparation*

A male Sprague Dawley rat was euthanized and then the hair of abdominal area was carefully removed with an electric clipper. A square section of the abdominal skin was excised. After incision, the adhering fat and other visceral debris in the skin were carefully removed from the undersurface with tweezers (2). The excised skin was used immediately. 

*Preparation of drug-EVA matrix containing an enhancer*

The drug-EVA matrix containing an enhancer was prepared by casting process. The weighed amount of EVA copolymer beads was dissolved in 20 mL of chloroform in a beaker, drug solution and enhancer (5%) were added with vigorous stirring. This mixture was poured onto a glass plate and the solvent was allowed to evaporate off at room temperature overnight. The drug content was calculated from the weight ratio of drug and copolymer. 

*HPLC determination of mexazolam*

Mexazolam was assayed by HPLC. The HPLC system consisted of a pump (Knauer, DE/K-120, U.S.A.), ultraviolet detector (Waters 484, U.S.A.), RESTEK C_18_ column (250 x 4.6mm, 5 ∞m), degaser, and an integrator (D520A, Youngin Scientific Co., Ltd., Korea). The mobile phase was composed of a mixture of acetonitrile and water (80:20 v/v). A flow rate of 1.0 mL/min yielded an operation pressure of ~1000 psi. The UV detector was operated at the wavelength of 242 nm. Under these conditions, the mexazolam peak appeared at a retention time of 5.7 min. 

*Permeation of mexazolam through the skin from EVA matrix containing an enhancer*

The enhancer might increase the fluidity of the stratum corneum and increase drug permeability through the rat skin. As enhancers, we used saturated fatty acid (capric acid, myristic acid, lauric acid, stearic acid, palmitic acid), unsaturated fatty acid (oleic acid and linoleic acid), pyrrolidones (2-pyrrolidone, *N*-methyl-2-pyrrolidone, poly vinyl pyrrolidone), propylene glycol derivatives (propylene glycol monolaurate, propylene glycol laurate, and propylene glycol monocaprylate), non-ionic surfactants (polyoxyethylene-2-oleyl ether, polyethylene-2-stearyl ether, polyoxyethylene-23-lauryl ether), and glycerides (oleyl macrogol-6 glycerides, caprylocaproyl macrogol-8 glycerides). The freshly excised, full-thickness skin sample was mounted on the receptor site of the diffusion cell with the stratum corneum side facing upwards into the donor compartment and the dermal side facing into the receptor compartment. A matrix was placed on the stratum corneum side, covered with a round glass plate, and clamped. 

The *in-vitro* release of mexazolam from the EVA matrix through rat skin was examined using a modified Keshary-Chien cell. Receptor medium, a 40% PEG 400 solution, was used to achieve sink conditions and maintained at 37°C by a circulating water bath. Total samples were withdrawn at predetermined times and immediately replaced by an equal volume of fresh medium. Permeation quantities of mexazolam were analyzed by HPLC at 242 nm. Each data point represents the average of three determinations. 

## Calculations

The permeation rate was calculated from the slope of the linear region of the permeation profile. The flux was calculated from the slope of the linear region of the Q versus t permeation profile. The cumulative amount of mexazolam through the rat skin was plotted against time (t). A linear profile was observed over 24 h and the slope of the linear portion of the curve was determined by linear regression. 

## Results and Discussion

*Solubility of mexazolam*

**Figure 1:**
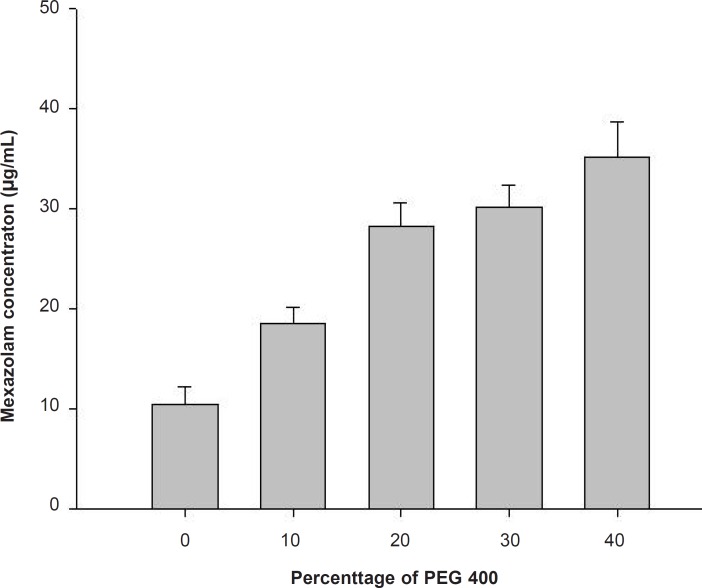
Solubility of mexazolam in PEG 400 according to the percentage volume of the solvent.

The aqueous solubility for mexazolam was extremely low and could be improved by addition of a water-miscible hydrophilic polymer like PEG 400. PEG 400 is an excellent solubilizer for many steroids (3), and here increased mexazolam solubility with increasing volume fractions (Figure 1). 

*Permeation studies through EVA membranes*

The cumulative amount of the drug permeating through a unit surface area (Q) can be expressed mathematically using the following relationship: 

$Q=P(C_{D}-C_{R)t})$ (1)

 where P is the permeability coefficient and C_D_ and C_R_ are the drug concentration in the donor (D) and the receptor (R) solutions, respectively. 

When the drug concentration in the donor solution (C_D_) is maintained at a level greater than the equilibrium solubility (Ce) of the drug (*i.e*., C_D_ > Ce) and the drug concentration in the receptor solution (C_R_) is maintained under the sink condition (*i.e*., C_R_ << Ce), Equation 1 can be simplified to:

$Q=\mathrm{P.}C_{e}\mathrm{.t}$ (2) 

to give a constant permeation profile. The rate of permeation is then defined by:

$\frac{Q}{t}=\mathrm{P.}C_{e}\mathrm{.t}$ (3)

As expected from Equation 2, when the mexazolam concentration in the donor solution was maintained at a level greater than its equilibrium solubility, a constant permeation profile was achieved. The rate of permeation (Q/t), which was measured from the slope of Q versus t plots (Equation 2), was increased with the addition of 40% (v/v) PEG 400. As expected from Equation 3, the permeation rate (Q/t) was increased based on the equilibrium solubility (C_e_) of mexazolam in the PEG 400 solutions. 

The effect of PEG 400 on the permeability coefficient (P) of mexazolam across the EVA membrane can be determined using Equation 4: 

$P=\frac{Q/t}{C_{s}}$ (4)

The permeability coefficient (P) decreased with increased volume fractions of PEG 400 in the saline solution. 

*Release of mexazolam from the EVA matrix *

A characteristic drug release profile of matrix-type drug delivery systems can be represented by Higuchi’s equation (4). The release from a system with dispersed drug in a homogeneous matrix should follow the relationship:

$Q=\left[D\left(2A-C_{s}\right)C_{s}\right]t^{1/2}$ (5) 

where Q is the amount of drug released after time τ per unit exposed area, D is the diffusivity of the drug in the matrix, A is the initial drug loading dose dispersed in the polymer matrix, C_s_ is the drug solubility in the matrix; D and C_s_ refer to diffusivity and solubility in the permeability field, respectively; τ is the tortuosity of the matrix, and ε is the porosity of the matrix. Although the two equations are for different mechanisms, they both describe drug release as being linear with the square root of time (6-9):

$Q=K_{H}.t^{1/2}$ (6)

where for the homogeneous matrix system:

$K_{H}={[D\left(2A-C_{s}\right]}^{1/2}$ (7) 

and for the granular matrix system

$K_{H}={[\frac{\mathrm{D\varepsilon}}{\tau }\left(2A-{\mathrm{\epsilon C}}_{s})C_{s}t\right]}^{1/2}$ (8)

The validity of the relationships has been confirmed experimentally using various systems (5, 10, and 11). 

*Effect s of drug loading dose*

**Figure 2:**
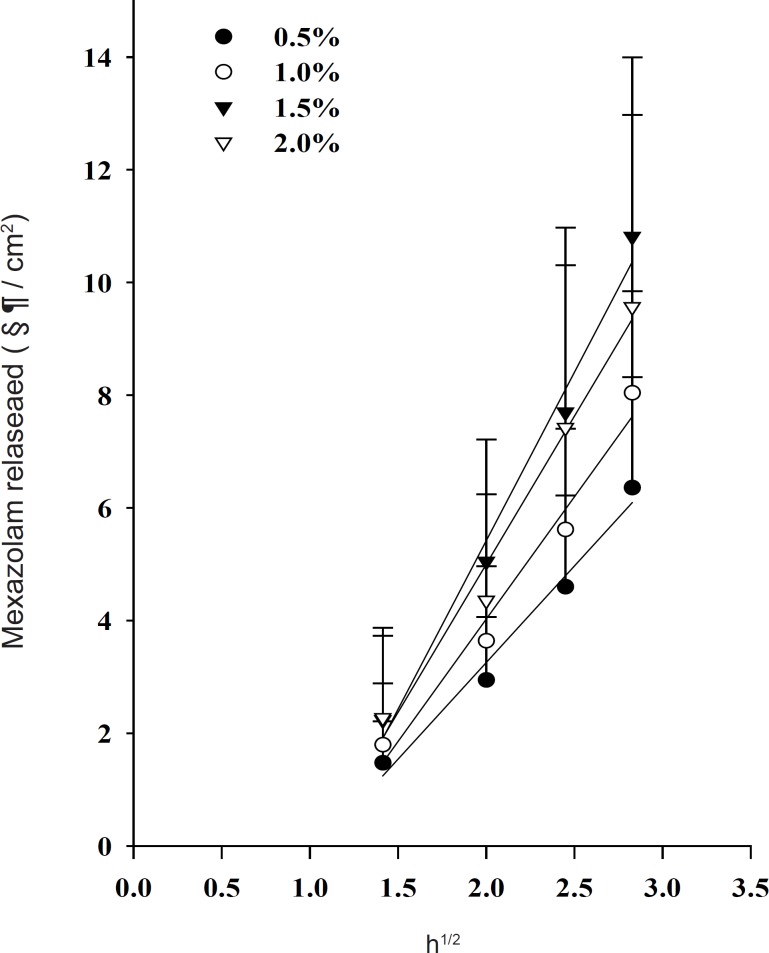
Effect of drug loading dose on the release of mexazolam from the EVA matrix at 37 °C.

**Figure 3:**
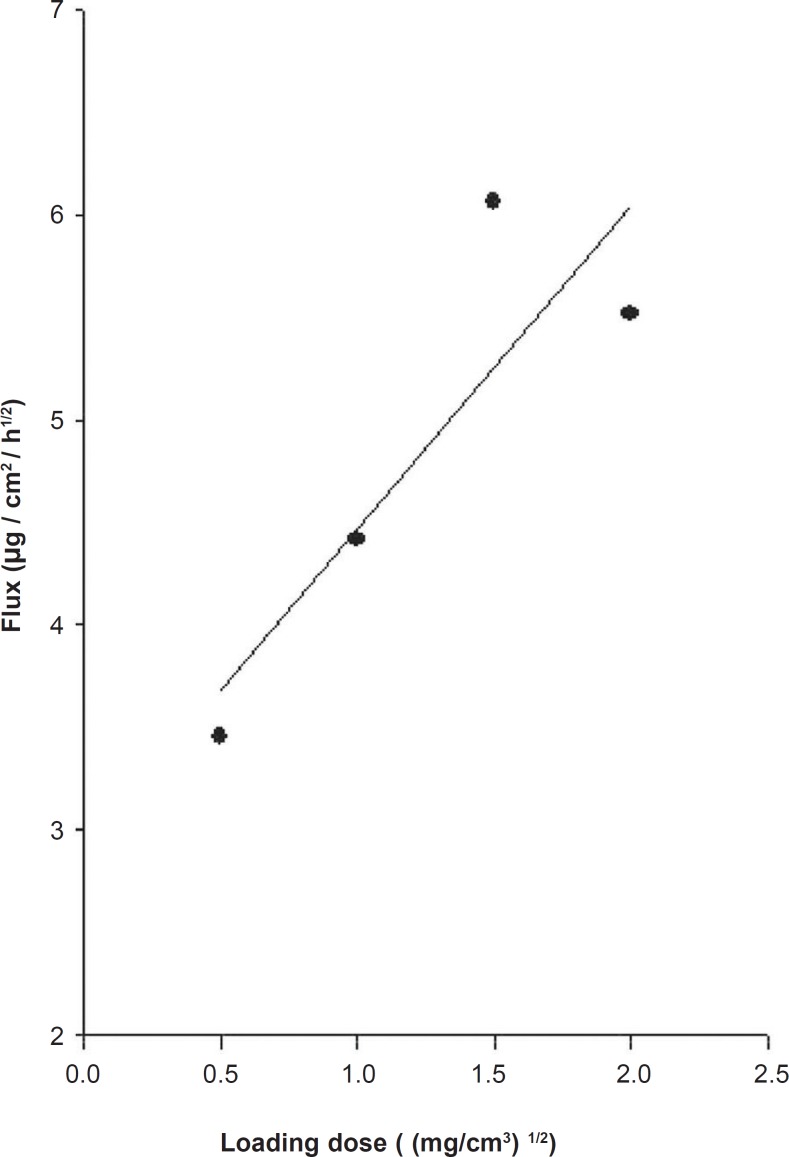
Relationship between mexazolam flux and drug loading dose in the EVA copolymer matrix at 37 °C; the PEG 400 volume fraction was maintained at 40% (v/v).

The release profiles of mexazolam from differently loaded EVA matrices over 8 h are shown in Figure 2. The plot of the cumulative amount of mexazolam released (Q) versus the square root of time (t^1/2^) shows a good linearity for all five different concentrations. As expected from Equation 6, 7, and 8, a plot of Q/t^1/2^ versus the square root of loading dose (A) yields a straight line. The Q/t^1/2^ increased proportionally to the increase in the square root of loaded dose of mexazolam (Figure 3).

*Effects of release media temperature*

**Figure 4:**
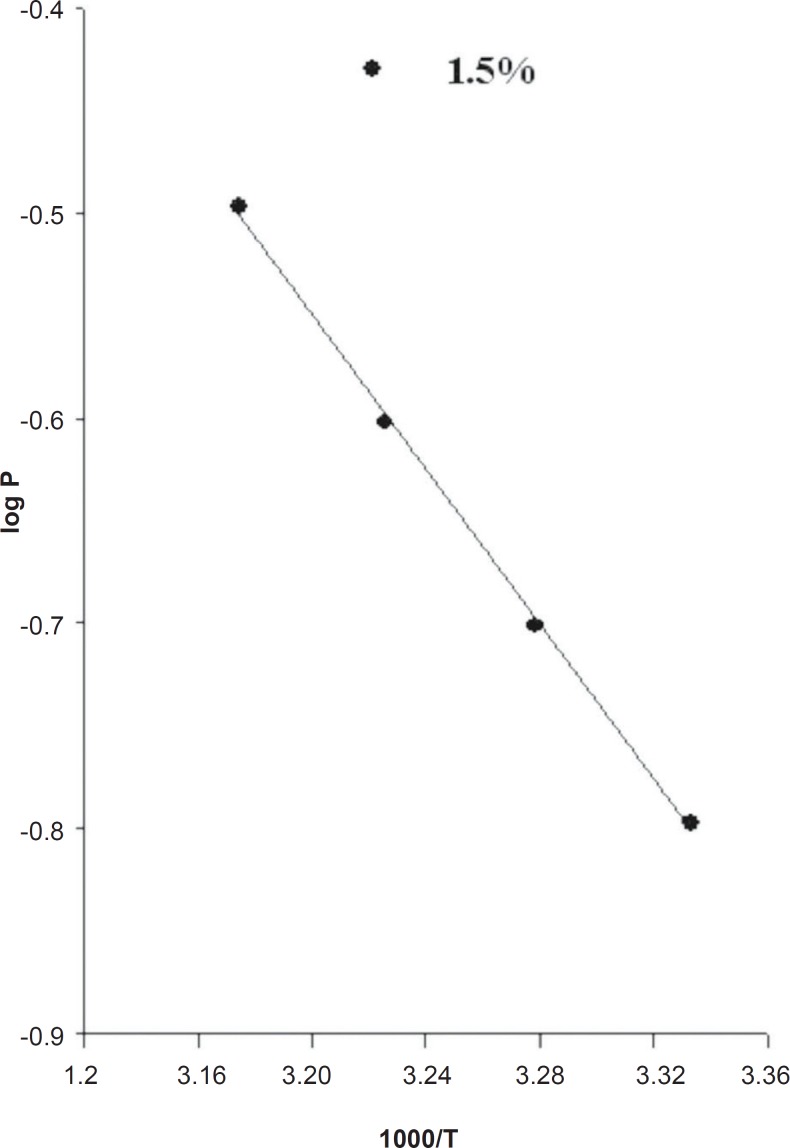
Effect of temperature on mexazolam release from the EVA matrix.

The dependency of the drug release profile on temperature is illustrated in Figure 4. The cumulative amount of the drug released (Q) was plotted as a function of the square root of time (t^1/2^). After an initial period of drug release, the release was approximately linear with respect to t^1/2^. The steady-state rate of drug release (Q/t½) was estimated from the slope of the linear Q - t^1/2 ^profile from 0 to 12 h. The drug release Q/t^1/2 ^values were increased by increasing temperature. In particular, the rate of drug release was increased approximately 1.65-fold by increasing the temperature of the system from 27 to 42°C. However, for practical uses, 37°C was chosen to reflect the temperature of the stratum corneum (12). 

This observation clearly indicates that the release of loratadine from the EVA matrix is an energy-linked process (13). The increase in release rate by increasing temperature suggests that the release characteristics of the copolymer would change over a range of body temperatures, and precautions should be taken for monitoring body temperature in practical applications. 

The permeability coefficient is then defined using the following equations: 

$P=\frac{\mathrm{FLUX}}{\mathrm{Solubility}}$ (9)

$P=P_{0}\mathrm{.e}-\frac{E_{a}}{\mathrm{RT}}$ (10)

Log P=Log P0-EaR . 2.303. 1000 . 1000T (11)

As expected from Equation 11, a plot of log P as a function of 1000/T yields a straight line (Figure 3).

$\mathrm{Slop}=-\frac{E_{a}}{\mathrm{R. 2.303}}.\frac{1}{1000}$ (12)

Ea =-Slope × R × 2.303 × 1000 cal =-slope× 1.987 ×2.303 kcal (13)

The activation energy (E_a_) for drug release from the EVA matrix, which was measured from slope of log P versus 1000/T plot, was 8.64 Kcal/mol for a 1.5% loading dose. 

*Effects of plasticizers on drug release from the EVA matrix*

Generally plasticizers increase the release of drugs by increasing chain mobility of the polymer. The plasticizer will interpose itself between the polymer chains and interact with the forces held together by extending and softening the polymer matrix (14). The plasticizer reduces the brittleness, improves flow, imparts flexibility, and increases toughness, strength, tear resistance, and impact resistance of the polymer. The selection of a suitable plasticizer and its concentration has a profound influence on mechanical properties and drug permeability (15). Increasing the amount of plasticizer could increase free film elongation and decrease tensile strength. A strong interaction between a drug and a polymer can significantly influence drug release through a polymeric film (16). The EVA matrix with citrate showed slightly increased drug release and with phthalate it showed dramatically increased rate of the release. Diethyl phthalate also increased the release rate of mexazolam. 

*Effects of enhancer on the permeation of mexazolam across the rats skin*

We evaluated the effects of enhancers on the skin permeation of mexazolam using a modified Keshary-Chien cell fitted with the intact excised rat skins. Skin permeation is a relatively slow process influenced by factors such as solubility, diffusion efficiency, and reaction rates. Improving the performance of the rate-limiting step in transdermal transport requires methods to quantify the contribution of particular pathways and to estimate key physico-chemical parameters (17). Nanosized carriers have improved drug permeation to deeper layers of the skin or systemic circulation. Some of the intrinsic ingredients in these systems, such as fatty acids, phospholipids, and surfactants, enhance penetration through the skin and increase drug absorption. New penetration enhancers have been developed to improve the percutaneous absorption of drugs. Certain combinations of enhancers (so called synergistic combinations) can deliver drugs but cause mild skin irritation,a frequent problem with many of the older enhancers (18). 

Fatty acids are currently receiving much attention as penetration enhancers (19) as they are an endogenous component of human skin. Fatty acids can differ in several features: chain length, characteristics of the double bonds (position, number, and configuration), branching schema, and substituents. These structural variations can influence their effects as skin penetration enhancers (20). Fatty acids are capable of inserting between the hydrophobic tails of the stratum corneum lipid bilayer, disturbing their packing, increasing their fluidity, and, subsequently, decreasing the diffusional resistance to permeants (21). Fatty acids (FAs) interact with intercellular lipid domains to promote the skin permeation of drugs with a wide range of polarities (22). The efficacy of FAs is intrinsically linked to their structure, with differences evident between saturated and unsaturated forms and different hydrocarbon chain lengths (19, 23). Unsaturated FAs, particularly those of *cis* conformation and C_18_ chain lengths, are more effective enhancers than their saturated counterparts, promoting the permeation of such penetrants as naloxone (24) and flurbiprofen (25). When introduced into the predominantly saturated, straight-chained lipid environment of the SC, these FAs intercalate and disrupt the ordered lipid array (26) and form separate fluid states that disorder endogenous lipids (27). Saturated fatty acids of linear shape and low solubility are less able to disrupt the lipid packing of the stratum corneum and to insert themselves into the lipid bilayers than kinked unsaturated fatty acids with high solubility. 

Table 1 shows the enhancement factor of enhancers such as saturated fatty acids, unsaturated fatty acids, the pyrrolidones, the propylene glycol derivatives, the glycerides, and the non-ionic surfactants. As a control, the mexazolam matrix without enhancers was also tested. For fatty acids, the unsaturated fatty acid group improved permeation more than the saturated fatty acid group. 

Surfactants enhance the permeability of drugs (28-33) through biological membranes, including skin (28) and increase the permeation rates of several drugs (24). Shin *et al.* recently studied the mechanism of the effect of non-ionic surfactants as permeation enhancers (31). Pre-treatment of the skin with the non-ionic surfactant has shown that the SC is loosely layered and intercellular spaces are wide (31, 33). Other experiments done in our laboratory showed that Brij 92 (polyoxyethylene 2-oleyl ether) was the best enhancing effect. Brij 35 (polyoxyethylene 2-stearyl ether) and Brij 72 (polyoxyethylene 23-lauryl ether) produced similar increases in permeation rate. 

**Table 1:** Enhancement factor of enhancers.

**Enhancer**	**Flux (µg/cm** ^2^ **/h)**	**EF**
Control	0.12 ± 0.03	1.00
polyoxyethylene 23-lauryl ether	0.14 ± 0.04	1.17
polyoxyethylene 2-stearyl ether	0.20 ± 0.03	1.67
polyoxyethylene 2-oleyl ether	0.15 ± 0.06	1.25
oleic acid	0.13 ± 0.04	1.10
linoleic acid	0.15 ± 0.03	1.25
caprylic acid	0.13 ± 0.03	1.10
lauric acid	0.13 ± 0.04	1.10
myristic acid	0.14 ± 0.13	1.17
oleoyl macrogol-6 glycerides	0.33 ± 0.04	2.75
caprylocaproyl macrogol-8 glycerides	0.18 ± 0.06	1.50
propylene glycol mono caprylate	0.18 ± 0.09	1.50
propylene glycol laurate	0.14 ± 0.09	1.17
propylene glycol monolaurate	0.20 ± 1.01	1.67
NMP	0.35 ± 0.04	2.91
2-pyrrolidone	0.13 ± 0.09	1.10
PVP	0.20 ± 0.06	1.67

Caprylocaproyl macrogol-glyceride (Labrasol) increased the passive transport of drug molecules. Oleoyl macrogo-6 glyceride (Labrafil) is a biocompatible and biodegradable PEG derivative (34) used as a co-surfactant in pharmaceutical systems such as microemulsions. Oleoyl macrogo-6 glyceride improved the mexazolam permeation rate. Propylene glycol (PG) is widely used as a vehicle for penetration enhancers and permeates well through human stratum corneum, and may carry drugs with it (35). The permeation of PG through tissue could alter thermodynamic activity of the drug in the vehicle, which would in turn modify the driving force for diffusion. PG may partition into the tissue, facilitating uptake of the drug into the skin and disrupting intercellular lipid packing within the stratum corneum bilayers (36). 

Pyrrolidones have been used as penetration enhancers in human skin for hydrophilic and lipophilic permeants (36). The pyrrolidones partition well into the human stratum corneum and may alter the solvent nature of the membrane to generate ‘reservoirs’ within skin membranes. Such a reservoir effect offers potential for sustained release of a permeant from the stratum corneum over an extended period (37). N-methyl-2-pyrrolidone showed the best enhancing effect (Table 1). 

## Conclusions

The release rate of drug from the EVA matrix increased with increased temperature and drug loading doses. The plasticizer, DEP, slightly increased the release of mexazolam. Among the many enhancers used, *N*-methyl-2-pyrrolidone showed the best enhancement factor. In conclusion, an EVA matrix containing plasticizer and permeation enhancer improved the transdermal delivery of mexazolam and may be useful in the development of a transdermal drug delivery system. 
